# Isotypic one-dimensional coordination polymers: *catena*-poly[[di­chlorido­cadmium]-μ-5,6-bis­(pyridin-2-yl)pyrazine-2,3-di­carboxyl­ato-κ^2^ *N* ^5^:*N* ^6^] and *catena*-poly[[di­chlorido­mercury(II)]-μ-5,6-bis­(pyridin-2-yl)pyrazine-2,3-di­carboxyl­ato-κ^2^ *N* ^5^:*N* ^6^]

**DOI:** 10.1107/S2056989016012093

**Published:** 2016-07-29

**Authors:** Montserrat Alfonso, Helen Stoeckli-Evans

**Affiliations:** a Institute of Chemistry, University of Neuchâtel, Av. de Bellevaux 51, CH-2000 Neuchâtel, Switzerland; b Insitute of Physics, University of Neuchâtel, rue Emile-Argand 11, CH-2000 Neuchâtel, Switzerland

**Keywords:** crystal structure, isotypic coordination polymer, one-dimensional, *M*N_2_Cl_2_ bisphenoidal coordination geometry, di­meth­yl ester, pyrazine, pyridine, C—H⋯Cl hydrogen bonding

## Abstract

The isotypic title one-dimensional coordination polymers, [CdCl_2_(C_18_H_14_N_4_O_4_)]_*n*_, (I), and [HgCl_2_(C_18_H_14_N_4_O_4_)]_*n*_, (II), are, respectively, the cadmium(II) and mercury(II) complexes of the dimethyl ester of 5,6-bis­(pyridin-2-yl)pyrazine-2,3-di­carb­oxy­lic acid. In both compounds, the metal ions are located on a twofold rotation axis and a second such axis bis­ects the C_ar_—C_ar_ bonds of the pyrazine ring. The metal ions are bridged by binding to the N atoms of the two pyridine rings and have an *M*N_2_Cl_2_ bisphenoidal coordination geometry. The metal–N_pyrazine_ distances are much longer than the metal–N_pyridine_ distances; the difference is 0.389 (2) Å for the Cd—N bonds but only 0.286 (5) Å for the Hg—N bond lengths. In the crystals of both compounds, the polymer chains are linked *via* pairs of C—H⋯Cl hydrogen bonds, forming corrugated slabs parallel to the *ac* plane.

## Abstract 2

The title complexes are isotypic one-dimensional coordination polymers. The metal ions are bridged by binding to the N atoms of the two pyridine rings, and have an *M*N_2_Cl_2_ bisphenoidal coordination geometry. In the crystals of both compounds, the polymer chains are linked *via* pairs of C—H⋯Cl hydrogen bonds, forming corrugated slabs parallel to the *ac* plane.

## Chemical context   

The crystal structures of the dimethyl and diethyl esters of 5,6-bis­(pyridin-2-yl)pyrazine-2,3-di­carb­oxy­lic acid (**L1H_2_**; Alfonso *et al.*, 2001 ▸) have been reported on recently (Alfonso & Stoeckli-Evans, 2016 ▸). They were originally synthesized to study the hydrolysis of these esters with first row transition metals (Alfonso, 1999 ▸). Subsequent studies of their reaction with *d* ^10^ or post-transition metals lead to the formation of the title compounds, and we report herein on the syntheses and crystal structures of the title isotypic cadmium(II) and mercury(II) coordination polymers.
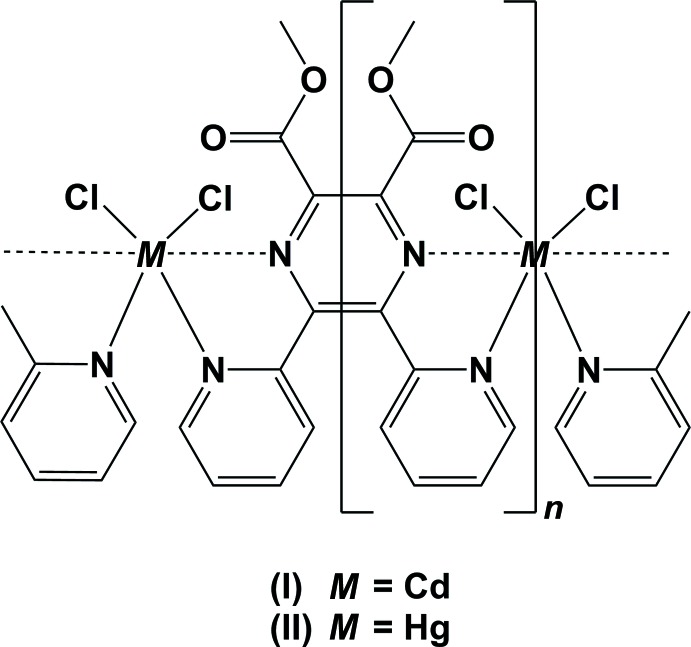


## Structural commentary   

**Figure 1:**
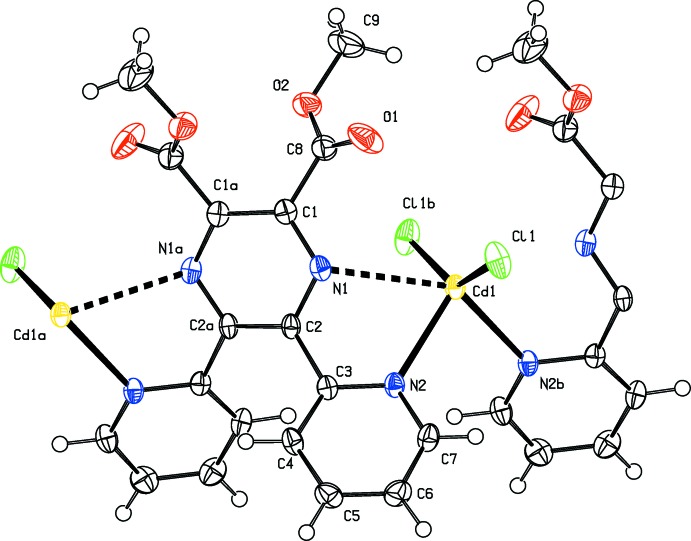
A view of the mol­ecular structure of compound (I), showing the atom labelling [symmetry codes: (*a*) −*x* + 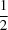, *y*, −*z* + 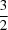; (*b*) −*x* + 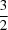, *y*, −*z* + 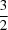]. Displacement ellipsoids are drawn at the 50% probability level

**Figure 2:**
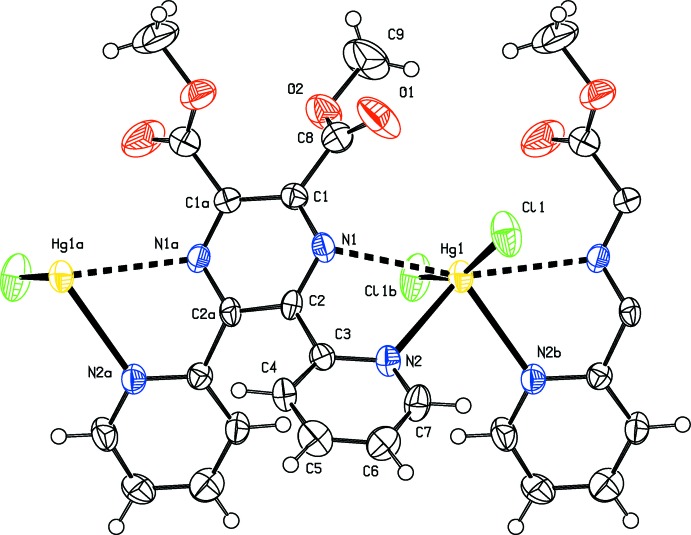
A view of the mol­ecular structure of compound (II), showing the atom labelling [symmetry codes: (*a*) −*x* + 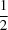, *y*, −*z* + 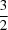; (*b*) −*x* + 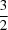, *y*, −*z* + 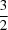]. Displacement ellipsoids are drawn at the 50% probability level.

In compounds (I) and (II), the metal atom is located on a twofold rotation axis and a second such axis bis­ects the C_ar_—C_ar_ bonds of the pyrazine ring; as illustrated in Fig. 1 ▸ for the cadmium complex (I), and in Fig. 2 ▸ for the mercury complex (II). Details of the bond lengths and bond angles involving the metal atoms are given in Table 1 ▸ for (I), and in Table 2 ▸ for (II). The metal atoms are bridged by binding to the N atoms of the two pyridine rings, N2 and N2^i^; Cd1—N2 = 2.3862 (17) Å in (I) and Hg1—N2 = 2.590 (5) Å in (II). The Cd1—Cl1 bonds [2.4137 (6) Å] are longer than the Hg1—Cl1 bonds [2.3464 (16) Å], while the reverse is true for the metal–N_pyridine_ bonds: Cd1—N2 [2.3862 (17) Å] is shorter than Hg1—N2 [2.590 (5) Å]. The link to the pyrazine N atoms, N2 and N2^i^, is much weaker: Cd1⋯N1 = 2.7757 (17) Å and Hg1⋯N1 = 2.876 (5) Å. The difference in the metal–N_pyrazine_ and metal–N_pyridine_ bond lengths is 0.389 (2) Å for the Cd—N bonds but only 0.286 (5) Å for the Hg—N bonds (see Tables 1 ▸ and 2 ▸).

**Figure 3:**
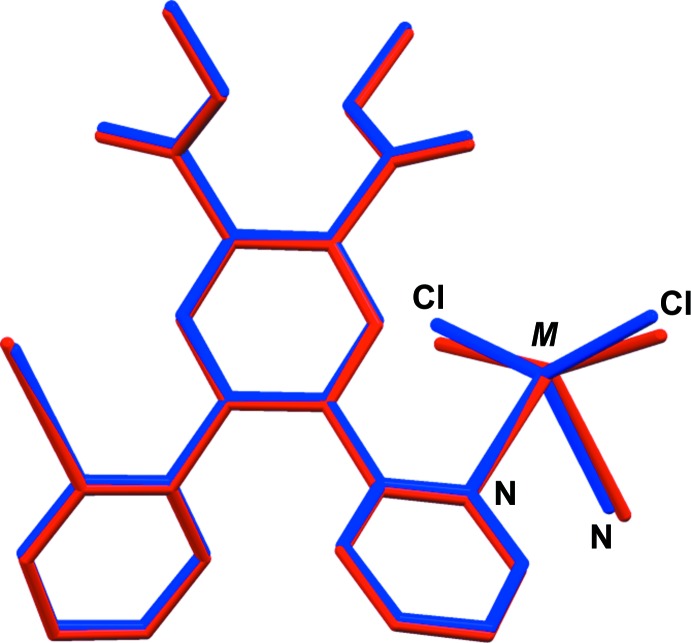
A view of the structural overlap of the cadmium complex (I) in blue and the mercury complex (II) in red; also illustrating the slight difference in the bisphenoidal coordination geometry of the two metal atoms (*M*N_2_Cl_2_).

The fourfold coordination geometry of the metal atoms differ slightly, as illustrated in Fig. 3 ▸ a structural overlap of the two compounds. In (I) atom Cd1 has a τ_4_ parameter of 0.53, while for the Hg1 atom in (II) the τ_4_ parameter = 0.30 (extreme values: τ_4_ = 0 for square-planar, 1 for tetra­hedral and 0.85 for trigonal–pyramidal geometry; Yang *et al.*, 2007 ▸). When also considering the values of the Cl—*M*—Cl and N—*M*—N bond angles in both compounds (see Tables 1 ▸ and 2 ▸), we conclude that both metal atoms (*M*) have a bisphenoidal *M*N_2_Cl_2_ coordination environment.

In both compounds, the pyrazine rings (N1/C1/C2/N1^i^/C1^i^/C2^i^) are not ideally planar [r.m.s. deviations are 0.096 and 0.092 Å for (I) and (II), respectively] and have twist-boat-like conformations [puckering parameters: amplitude (*Q*) = 0.166 (2) Å, θ = 87.8 (7)°, φ = 270.0 (7)° for (I), and amplitude (*Q*) = 0.160 (6) Å, θ = 90 (2)°, φ = 270 (2)° for (II); symmetry code: (i) −*x* + 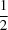, *y*, −*z* + 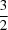].

The pyridine rings (N2/C3–C7), are inclined to the pyrazine ring mean planes by 40.58 (10)° in (I) and 42.1 (3)° in (II), and to one another by 67.37 (10)° in (I) and 67.3 (3)° in (II). The methyl­carboxyl­ate groups (C9/O2/C8/O1) are planar to within 0.019 (2) Å for atom O2 in (I) and 0.20 (7) Å for atom C8 in (II). Their mean planes are inclined to the mean plane of the pyrazine ring and to one another by 44.44 (16) and 68.8 (2)°, respectively, in (I), and by 43.0 (3) and 75.7 (5)°, respectively, in (II).

**Figure 4:**
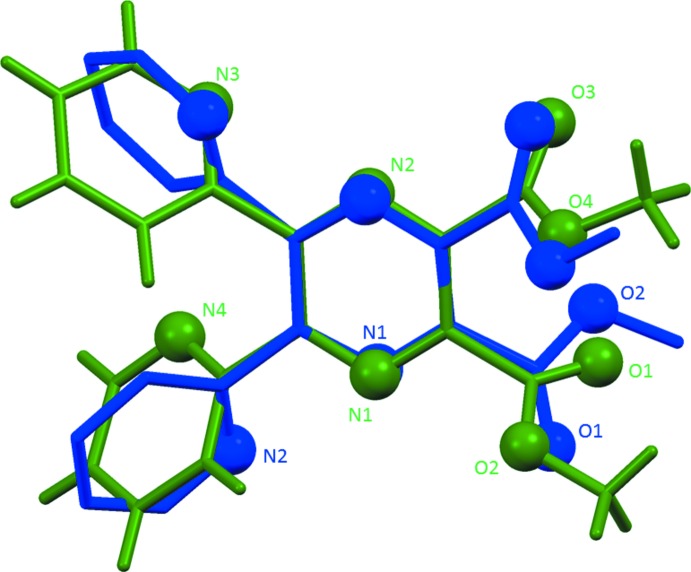
A view of the structural overlap of the ligand (**Me_2_L**, green; Alfonso & Stoeckli-Evans, 2016 ▸) and the coordinating ligand (blue) in compound (I).

It can be seen from Fig. 4 ▸, a structural overlap of the ligand itself (Alfonso & Stoeckli-Evans, 2016 ▸) with the coordinating ligand in compound (I), that both the pyridine ring involving atom N4, and the carboxyl­ate group, involving atoms O1 and O2, have been rotated by *ca* 100 and 160°, respectively, on coordination to the metal atom. While the pyrazine ring is ideally planar in the ligand (r.m.s. deviation = 0.032 Å), on coordination it is less planar with r.m.s. deviations of 0.096 and 0.092 Å for (I) and (II), respectively.

## Supra­molecular features   

**Figure 5:**
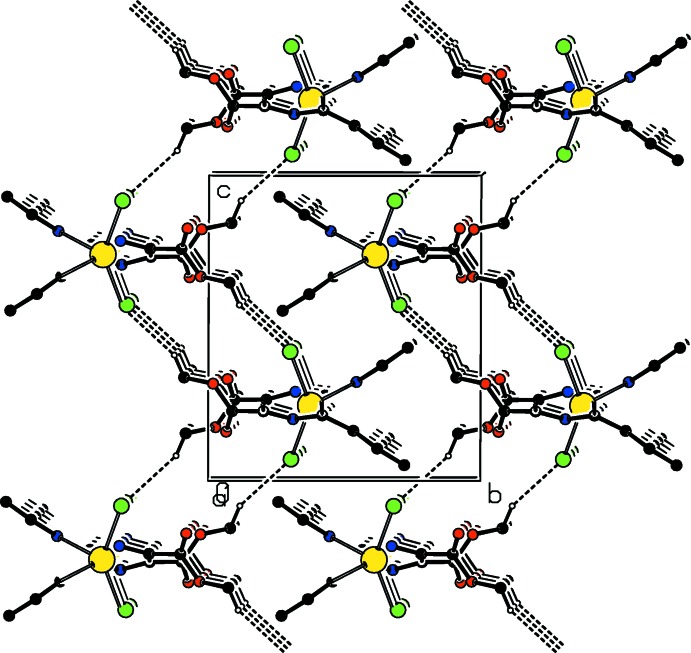
A view along the *a* axis of the crystal packing of compound (I). The hydrogen bonds are shown as dashed lines (see Table 3 ▸; only H atom H9*A* has been included).

**Figure 6:**
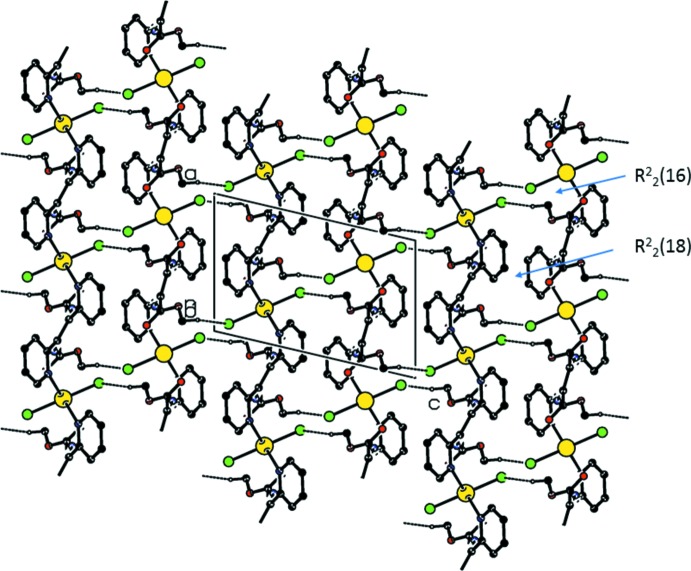
A projection along the *b* axis of the crystal packing of compound (II). The hydrogen bonds are shown as dashed lines (see Table 4 ▸; only H atom H9*A* has been included).

In the crystals of both compounds, the polymer chains are linked *via* a pair of C—H⋯Cl hydrogen bonds, forming corrugated slabs parallel to the *ac* plane, as illustrated in Fig. 5 ▸. Within the slabs, the hydrogen bonding forms 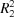
(16) and 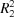
(18) type loops, as shown in Fig. 6 ▸. Details of the hydrogen bonding are given in Table 3 ▸ for compound (I) and Table 4 ▸ for compound (II). There are no other significant inter­molecular inter­actions present for either structure.

## Database survey   

A search of the Cambridge Structural Database (Version 5.37, update May 2016; Groom *et al.*, 2016 ▸) for *M*N_2_Cl_2_ (where *M* = Cd and Hg; N_pyridine_) four-coordinate metal ions yielded eight hits for *M* = cadmium and 52 hits for *M* = mercury. For the cadmium complexes, the Cd—Cl bonds are consistently longer than the Cd—N_pyridine_ bonds, and the Cl—Cd—Cl bond angles are consistently larger than the N—Cd—N bond angle, as in compound (I). A good example is di­chloridobis­{2-[(tri­phenyl­meth­yl)amino]­pyridine-κ*N*}cadmium (VIWKIW; Zhang, 2008 ▸), with approximate bond lengths and bond angles of Cd—Cl = 2.387, Cd—N = 2.285 Å, Cl—Cd—Cl = 121.2 and N—Cd—N = 95.2 °.

For the mercury complexes, the Hg—Cl bond lengths are either longer or shorter than the Hg—N_pyridine_ bond lengths. For example, in bis­(2-amino-3-methyl­pyridine)­dichlorido­mercury(II) (LEHMAO; Tadjarodi *et al.*, 2012 ▸) the approximate bond lengths and angles are Hg—Cl = 2.452, Hg—N = 2.267 Å, Cl—Hg—Cl = 119.9 and N—Hg—N = 101.3°, while in di­chlorido­bis­(3,3,3′,3′-tetra­methyl-2,2′,3,3′-tetra­hydro-1,1′-spiro­bi[indene]6,6′-diyl diisonicotinato)mercury (HUKTAJ; Lin *et al.*, 2010 ▸) the approximate bond lengths and angles are Hg—Cl = 2.345, Hg—N = 2.593 Å, Cl—Hg—Cl = 167.5 and N—Hg—N = 104.7°. This latter example is similar to the situation in compound (II).

## Synthesis and crystallization   

The synthesis of the ligand dimethyl-5,6-bis­(pyridin-2-yl)pyrazine-2,3-di­carboxyl­ate (**Me_2_L**) has been reported on recently (Alfonso & Stoeckli-Evans, 2016 ▸).

**Synthesis of compound (I)**: CdCl_2_·2H_2_O (22 mg, 0.1 mmol) in 25 ml of dry MeOH was slowly added to a solution of **Me_2_L** in 10 ml of dry MeOH. The colourless solution that formed was stirred at room temperature for 1 h, then filtered to remove any impurities. The filtrate was allowed to stand over several days until colourless square rod-like crystals were obtained (yield: 40 mg, 75%). Elemental analysis for C_18_H_14_N_4_CdCl_2_O_4_ (*Mw* = 533.63); calculated C 40.51, H 2.64, N 10.50%; found C 40.49, H 2.53, N 10.59%. Selected IR bands (KBr pellet, cm^−1^): ν = 3066(*w*), 2997(*w*), 1751(*vs*), 1593(*m*), 1568(*w*), 1549(*w*), 1479(*m*), 1450(*m*), 1403(*m*), 1339(*s*), 1297(*m*), 1273(*m*), 1261(*m*), 1228(*s*), 1193(*m*), 1177(*m*), 1162(*m*), 1120(*m*), 1109(*w*), 1088(*s*), 1009(*m*), 974(*m*), 918(*w*), 827(*m*), 803(*m*), 789(*m*), 770(*w*), 758(*m*), 554(*m*).

**Synthesis of compound (II)**: **Me_2_L** (35 mg, 0.1 mmol) was added in solid form to a solution of HgCl_2_·2H_2_O (30 mg, 0.1 mmol) in 25 ml of dry MeOH. The colourless solution immediately obtained was stirred at room temperature for 2 h, filtered to remove any impurity, and the filtrate allowed to evaporate slowly. After two days colourless needle-like crystals were obtained (yield: 47mg, 76%). Elemental analysis for C_18_H_14_N_4_Cl_2_HgO_4_ (*Mw* = 621.82); calculated C 34.77, H 2.27, N 9.01%; found C 34.79, H 2.44, N 9.03%. Selected IR bands (KBr pellet, cm^−1^): ν = 3065(*w*), 2997(*w*), 2951(*w*), 2882(*w*), 1747(*vs*), 1590(*m*), 1568(*m*), 1546(*w*), 1477(*m*), 1447(*m*), 1402(*m*), 1338(*s*), 1279(*m*), 1223(*s*), 1195(*m*), 1176(*s*), 1105(*w*), 1086(*s*), 1003(*m*), 973(*w*), 827(*w*), 802(*m*), 788(*m*), 769(*m*), 755(*m*), 553(*m*).

## Refinement   

Crystal data, data collection and structure refinement details are summarized in Table 5 ▸. For both compounds the C-bound H atoms were included in calculated positions and treated as riding atoms: C—H = 0.93–0.97 Å with *U*_iso_(H) = 1.5*U*_eq_(C-meth­yl) and 1.2*U*_eq_(C) for other H-atoms. For the mercury complex (II), *R*_int_ = 0.000 as only one equivalent was measured.

## Supplementary Material

Crystal structure: contains datablock(s) I, II, Global. DOI: 10.1107/S2056989016012093/zl2672sup1.cif

Structure factors: contains datablock(s) I. DOI: 10.1107/S2056989016012093/zl2672Isup2.hkl

Structure factors: contains datablock(s) II. DOI: 10.1107/S2056989016012093/zl2672IIsup3.hkl

CCDC references: 1495931, 1495930

Additional supporting information:  crystallographic information; 3D view; checkCIF report

## Figures and Tables

**Table 1:** Selected geometric parameters (Å, °) for (I)

Cd1—Cl1	2.4137 (6)	Cd1—N1	2.7757 (17)
Cd1—N2	2.3862 (17)		
			
Cl1^i^—Cd1—Cl1	142.43 (3)	N2—Cd1—Cl1^i^	110.87 (4)
N2—Cd1—N2^i^	85.02 (8)	N2—Cd1—Cl1	96.81 (4)

Symmetry code: (i) 
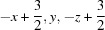
.

**Table 2:** Selected geometric parameters (Å, °) for (II)

Hg1—Cl1	2.3464 (16)	Hg1—N1	2.876 (5)
Hg1—N2	2.590 (5)		
			
Cl1^i^—Hg1—Cl1	158.87 (12)	Cl1^i^—Hg1—N2	102.86 (12)
N2—Hg1—N2^i^	83.1 (2)	Cl1—Hg1—N2	92.97 (12)

Symmetry code: (i) 
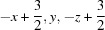
.

**Table 3:** Hydrogen-bond geometry (Å, °) for (I)

*D*—H⋯*A*	*D*—H	H⋯*A*	*D*⋯*A*	*D*—H⋯*A*
C9—H9*A*⋯Cl1^ii^	0.97	2.69	3.577 (3)	151

Symmetry code: (ii) 
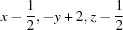
.

**Table 4:** Hydrogen-bond geometry (Å, °) for (II)

*D*—H⋯*A*	*D*—H	H⋯*A*	*D*⋯*A*	*D*—H⋯*A*
C9—H9*A*⋯Cl1^ii^	0.96	2.81	3.647 (9)	146

Symmetry code: (ii) 
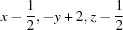
.

**Table 5:** Experimental details

	(I)	(II)
Crystal data		
Chemical formula	[CdCl_2_(C_18_H_14_N_4_O_4_)]	[HgCl_2_(C_18_H_14_N_4_O_4_)]
*M*_r_	533.63	621.82
Crystal system, space group	Monoclinic, *P*2/*n*	Monoclinic, *P*2/*n*
Temperature (K)	223	293
*a*, *b*, *c* (Å)	7.8919 (7), 10.5898 (7), 12.0875 (12)	8.1042 (6), 10.6002 (16), 12.2063 (10)
β (°)	102.061 (11)	103.158 (7)
*V* (Å^3^)	987.90 (15)	1021.07 (19)
*Z*	2	2
Radiation type	Mo *K*α	Mo *K*α
μ (mm^−1^)	1.41	7.83
Crystal size (mm)	0.40 × 0.20 × 0.10	0.49 × 0.23 × 0.04

Data collection		
Diffractometer	Stoe IPDS 1 image-plate	Stoe–Siemens AED2 four-circle
Absorption correction	Multi-scan (*MULABS*; Spek, 2009 ▸)	ψ scan (*X-RED*; Stoe & Cie, 1997 ▸)
*T*_min_, *T*_max_	0.938, 1.000	0.319, 1.000
No. of measured, independent and observed [*I* > 2σ(*I*)] reflections	7196, 1918, 1684	1855, 1855, 1723
*R*_int_	0.037	0.000
(sin θ/λ)_max_ (Å^−1^)	0.614	0.600

Refinement		
*R*[*F* ^2^ > 2σ(*F* ^2^)], *wR*(*F* ^2^), *S*	0.022, 0.050, 0.95	0.032, 0.074, 1.11
No. of reflections	1918	1855
No. of parameters	133	133
H-atom treatment	H-atom parameters constrained	H-atom parameters constrained
Δρ_max_, Δρ_min_ (e Å^−3^)	0.34, −0.64	1.23, −1.08

Computer programs: *EXPOSE*, *CELL* and *INTEGRATE* in *IPDS-I* (Stoe & Cie, 2004 ▸), *STADI4* and *X-RED* (Stoe & Cie, 1997 ▸), *SHELXS97* (Sheldrick, 2008 ▸), *SHELXL2014* (Sheldrick, 2015 ▸), *Mercury* (Macrae *et al.*, 2008 ▸), *PLATON* (Spek, 2009 ▸) and *publCIF* (Westrip, 2010 ▸).

## supplementary crystallographic information

### (I) catena-Poly[[dichloridocadmium(II)]-µ-5,6-bis(pyridin-2-yl)pyrazine-2,3-dicarboxylato-κ2N5:N6] Crystal data

**Table:**

[CdCl_2_(C_18_H_14_N_4_O_4_)]	*F*(000) = 528
*M**_r_* = 533.63	*D*_x_ = 1.794 Mg m^−^^3^
Monoclinic, *P*2/*n*	Mo *K*α radiation, λ = 0.71073 Å
*a* = 7.8919 (7) Å	Cell parameters from 5000 reflections
*b* = 10.5898 (7) Å	θ = 2.0–25.9°
*c* = 12.0875 (12) Å	µ = 1.41 mm^−^^1^
β = 102.061 (11)°	*T* = 223 K
*V* = 987.90 (15) Å^3^	Plate, colourless
*Z* = 2	0.40 × 0.20 × 0.10 mm

### (I) catena-Poly[[dichloridocadmium(II)]-µ-5,6-bis(pyridin-2-yl)pyrazine-2,3-dicarboxylato-κ2N5:N6] Data collection

**Table:**

Stoe IPDS 1 image-plate diffractometer	1918 independent reflections
Radiation source: fine-focus sealed tube	1684 reflections with *I* > 2σ(*I*)
Plane graphite monochromator	*R*_int_ = 0.037
φ rotation scans	θ_max_ = 25.9°, θ_min_ = 2.6°
Absorption correction: multi-scan (MULABS; Spek, 2009)	*h* = −9→8
*T*_min_ = 0.938, *T*_max_ = 1.000	*k* = −12→12
7196 measured reflections	*l* = −14→14

### (I) catena-Poly[[dichloridocadmium(II)]-µ-5,6-bis(pyridin-2-yl)pyrazine-2,3-dicarboxylato-κ2N5:N6] Refinement

**Table:**

Refinement on *F*^2^	Primary atom site location: structure-invariant direct methods
Least-squares matrix: full	Secondary atom site location: difference Fourier map
*R*[*F*^2^ > 2σ(*F*^2^)] = 0.022	Hydrogen site location: inferred from neighbouring sites
*wR*(*F*^2^) = 0.050	H-atom parameters constrained
*S* = 0.95	*w* = 1/[σ^2^(*F*_o_^2^) + (0.0331*P*)^2^] where *P* = (*F*_o_^2^ + 2*F*_c_^2^)/3
1918 reflections	(Δ/σ)_max_ < 0.001
133 parameters	Δρ_max_ = 0.34 e Å^−^^3^
0 restraints	Δρ_min_ = −0.64 e Å^−^^3^

### (I) catena-Poly[[dichloridocadmium(II)]-µ-5,6-bis(pyridin-2-yl)pyrazine-2,3-dicarboxylato-κ2N5:N6] Special details

**Table:**

Geometry. All esds (except the esd in the dihedral angle between two l.s. planes) are estimated using the full covariance matrix. The cell esds are taken into account individually in the estimation of esds in distances, angles and torsion angles; correlations between esds in cell parameters are only used when they are defined by crystal symmetry. An approximate (isotropic) treatment of cell esds is used for estimating esds involving l.s. planes.

### (I) catena-Poly[[dichloridocadmium(II)]-µ-5,6-bis(pyridin-2-yl)pyrazine-2,3-dicarboxylato-κ2N5:N6] Fractional atomic coordinates and isotropic or equivalent isotropic displacement parameters (Å^2^)

**Table:**

	*x*	*y*	*z*	*U*_iso_*/*U*_eq_	
Cd1	0.7500	0.62102 (2)	0.7500	0.02022 (8)	
Cl1	0.91939 (8)	0.69442 (6)	0.92817 (4)	0.03420 (15)	
O1	0.5767 (3)	0.92856 (19)	0.83392 (17)	0.0493 (5)	
O2	0.3843 (2)	0.97480 (15)	0.67432 (14)	0.0320 (4)	
N1	0.4264 (2)	0.68120 (17)	0.79108 (14)	0.0207 (4)	
N2	0.6038 (2)	0.45491 (16)	0.82537 (14)	0.0200 (4)	
C1	0.3400 (3)	0.7889 (2)	0.76437 (17)	0.0212 (4)	
C2	0.3377 (3)	0.5727 (2)	0.77768 (16)	0.0186 (4)	
C3	0.4350 (3)	0.45962 (19)	0.82904 (15)	0.0183 (4)	
C4	0.3567 (3)	0.3710 (2)	0.88644 (17)	0.0235 (4)	
H4	0.2384	0.3774	0.8878	0.028*	
C5	0.4558 (3)	0.2725 (2)	0.94191 (18)	0.0280 (5)	
H5	0.4058	0.2111	0.9813	0.034*	
C6	0.6287 (3)	0.2667 (2)	0.93806 (19)	0.0292 (5)	
H6	0.6987	0.2007	0.9742	0.035*	
C7	0.6976 (3)	0.3592 (2)	0.88040 (18)	0.0254 (5)	
H7	0.8163	0.3552	0.8794	0.030*	
C8	0.4490 (3)	0.9051 (2)	0.7648 (2)	0.0277 (5)	
C9	0.4881 (4)	1.0825 (3)	0.6553 (3)	0.0502 (8)	
H9A	0.4411	1.1183	0.5814	0.075*	
H9B	0.6065	1.0555	0.6587	0.075*	
H9C	0.4861	1.1456	0.7131	0.075*	

### (I) catena-Poly[[dichloridocadmium(II)]-µ-5,6-bis(pyridin-2-yl)pyrazine-2,3-dicarboxylato-κ2N5:N6] Atomic displacement parameters (Å^2^)

**Table:**

	*U*^11^	*U*^22^	*U*^33^	*U*^12^	*U*^13^	*U*^23^
Cd1	0.01561 (12)	0.02520 (13)	0.01861 (12)	0.000	0.00071 (8)	0.000
Cl1	0.0255 (3)	0.0559 (4)	0.0204 (3)	−0.0113 (3)	0.0031 (2)	−0.0094 (2)
O1	0.0389 (11)	0.0448 (11)	0.0538 (12)	−0.0200 (9)	−0.0140 (9)	0.0170 (9)
O2	0.0327 (9)	0.0284 (8)	0.0340 (9)	−0.0013 (7)	0.0045 (7)	0.0099 (7)
N1	0.0177 (9)	0.0279 (9)	0.0163 (8)	−0.0020 (8)	0.0032 (7)	0.0030 (7)
N2	0.0157 (9)	0.0263 (9)	0.0177 (8)	0.0006 (7)	0.0025 (7)	0.0027 (7)
C1	0.0200 (11)	0.0264 (11)	0.0165 (10)	−0.0007 (9)	0.0023 (8)	0.0011 (8)
C2	0.0152 (11)	0.0263 (10)	0.0151 (9)	0.0007 (9)	0.0052 (8)	0.0016 (8)
C3	0.0166 (11)	0.0243 (10)	0.0132 (9)	−0.0014 (8)	0.0010 (8)	−0.0014 (8)
C4	0.0172 (10)	0.0323 (11)	0.0204 (10)	−0.0041 (10)	0.0023 (8)	0.0027 (9)
C5	0.0304 (13)	0.0296 (12)	0.0232 (11)	−0.0044 (10)	0.0037 (9)	0.0076 (9)
C6	0.0297 (13)	0.0295 (12)	0.0258 (12)	0.0050 (10)	0.0000 (10)	0.0069 (9)
C7	0.0189 (11)	0.0331 (13)	0.0235 (11)	0.0046 (9)	0.0030 (9)	0.0033 (9)
C8	0.0253 (13)	0.0280 (12)	0.0295 (12)	−0.0001 (9)	0.0049 (10)	0.0069 (9)
C9	0.0538 (19)	0.0375 (15)	0.0599 (18)	−0.0097 (13)	0.0134 (15)	0.0201 (13)

### (I) catena-Poly[[dichloridocadmium(II)]-µ-5,6-bis(pyridin-2-yl)pyrazine-2,3-dicarboxylato-κ2N5:N6] Geometric parameters (Å, º)

**Table:**

Cd1—Cl1^i^	2.4137 (6)	C2—C2^ii^	1.406 (4)
Cd1—Cl1	2.4137 (6)	C2—C3	1.486 (3)
Cd1—N2	2.3862 (17)	C3—C4	1.387 (3)
Cd1—N2^i^	2.3862 (17)	C4—C5	1.389 (3)
Cd1—N1	2.7757 (17)	C4—H4	0.9400
O1—C8	1.193 (3)	C5—C6	1.377 (4)
O2—C8	1.330 (3)	C5—H5	0.9400
O2—C9	1.450 (3)	C6—C7	1.378 (3)
N1—C1	1.333 (3)	C6—H6	0.9400
N1—C2	1.337 (3)	C7—H7	0.9400
N2—C3	1.343 (3)	C9—H9A	0.9700
N2—C7	1.346 (3)	C9—H9B	0.9700
C1—C1^ii^	1.390 (4)	C9—H9C	0.9700
C1—C8	1.501 (3)		
			
Cl1^i^—Cd1—Cl1	142.43 (3)	C3—C4—H4	120.5
N2—Cd1—N2^i^	85.02 (8)	C5—C4—H4	120.5
N2—Cd1—Cl1^i^	110.87 (4)	C6—C5—C4	118.7 (2)
N2^i^—Cd1—Cl1^i^	96.81 (4)	C6—C5—H5	120.7
N2—Cd1—Cl1	96.81 (4)	C4—C5—H5	120.7
N2^i^—Cd1—Cl1	110.87 (4)	C5—C6—C7	118.9 (2)
C8—O2—C9	115.7 (2)	C5—C6—H6	120.5
C1—N1—C2	118.54 (18)	C7—C6—H6	120.5
C3—N2—C7	117.28 (18)	N2—C7—C6	123.4 (2)
C3—N2—Cd1	123.17 (13)	N2—C7—H7	118.3
C7—N2—Cd1	118.96 (14)	C6—C7—H7	118.3
N1—C1—C1^ii^	120.30 (13)	O1—C8—O2	125.7 (2)
N1—C1—C8	115.93 (19)	O1—C8—C1	124.9 (2)
C1^ii^—C1—C8	123.75 (12)	O2—C8—C1	109.33 (19)
N1—C2—C2^ii^	119.71 (12)	O2—C9—H9A	109.5
N1—C2—C3	115.48 (17)	O2—C9—H9B	109.5
C2^ii^—C2—C3	124.75 (12)	H9A—C9—H9B	109.5
N2—C3—C4	122.68 (18)	O2—C9—H9C	109.5
N2—C3—C2	116.45 (17)	H9A—C9—H9C	109.5
C4—C3—C2	120.60 (19)	H9B—C9—H9C	109.5
C3—C4—C5	119.0 (2)		
			
C2—N1—C1—C1^ii^	7.2 (3)	C2—C3—C4—C5	−174.32 (19)
C2—N1—C1—C8	−171.29 (18)	C3—C4—C5—C6	0.1 (3)
C1—N1—C2—C2^ii^	8.5 (3)	C4—C5—C6—C7	0.6 (3)
C1—N1—C2—C3	−168.82 (18)	C3—N2—C7—C6	0.8 (3)
C7—N2—C3—C4	0.0 (3)	Cd1—N2—C7—C6	172.27 (17)
Cd1—N2—C3—C4	−171.05 (15)	C5—C6—C7—N2	−1.2 (3)
C7—N2—C3—C2	174.10 (18)	C9—O2—C8—O1	5.2 (4)
Cd1—N2—C3—C2	3.0 (2)	C9—O2—C8—C1	−172.5 (2)
N1—C2—C3—N2	−36.1 (2)	N1—C1—C8—O1	−41.0 (3)
C2^ii^—C2—C3—N2	146.7 (2)	C1^ii^—C1—C8—O1	140.6 (3)
N1—C2—C3—C4	138.1 (2)	N1—C1—C8—O2	136.7 (2)
C2^ii^—C2—C3—C4	−39.0 (3)	C1^ii^—C1—C8—O2	−41.7 (3)
N2—C3—C4—C5	−0.5 (3)		

Symmetry codes: (i) −*x*+3/2, *y*, −*z*+3/2; (ii) −*x*+1/2, *y*, −*z*+3/2.

### (I) catena-Poly[[dichloridocadmium(II)]-µ-5,6-bis(pyridin-2-yl)pyrazine-2,3-dicarboxylato-κ2N5:N6] Hydrogen-bond geometry (Å, º)

**Table:**

*D*—H···*A*	*D*—H	H···*A*	*D*···*A*	*D*—H···*A*
C9—H9*A*···Cl1^iii^	0.97	2.69	3.577 (3)	151

Symmetry code: (iii) *x*−1/2, −*y*+2, *z*−1/2.

### (II) catena-Poly[[dichloridomercury(II)]-µ-5,6-bis(pyridin-2-yl)pyrazine-2,3-dicarboxylato-κ2N5:N6] Crystal data

**Table:**

[HgCl_2_(C_18_H_14_N_4_O_4_)]	*F*(000) = 592
*M**_r_* = 621.82	*D*_x_ = 2.023 Mg m^−^^3^
Monoclinic, *P*2/*n*	Mo *K*α radiation, λ = 0.71073 Å
*a* = 8.1042 (6) Å	Cell parameters from 31 reflections
*b* = 10.6002 (16) Å	θ = 12.5–15.9°
*c* = 12.2063 (10) Å	µ = 7.83 mm^−^^1^
β = 103.158 (7)°	*T* = 293 K
*V* = 1021.07 (19) Å^3^	Plate, colourless
*Z* = 2	0.49 × 0.23 × 0.04 mm

### (II) catena-Poly[[dichloridomercury(II)]-µ-5,6-bis(pyridin-2-yl)pyrazine-2,3-dicarboxylato-κ2N5:N6] Data collection

**Table:**

Stoe–Siemens AED2 four-circle diffractometer	1723 reflections with *I* > 2σ(*I*)
Radiation source: fine-focus sealed tube	*R*_int_ = 0.000
Plane graphite monochromator	θ_max_ = 25.2°, θ_min_ = 2.6°
ω/\2q scans	*h* = −9→9
Absorption correction: ψ scan (X-RED software; Stoe & Cie, 1997)	*k* = 0→12
*T*_min_ = 0.319, *T*_max_ = 1.000	*l* = 0→14
1855 measured reflections	2 standard reflections every 60 min
1855 independent reflections	intensity decay: 2%

### (II) catena-Poly[[dichloridomercury(II)]-µ-5,6-bis(pyridin-2-yl)pyrazine-2,3-dicarboxylato-κ2N5:N6] Refinement

**Table:**

Refinement on *F*^2^	Primary atom site location: structure-invariant direct methods
Least-squares matrix: full	Secondary atom site location: difference Fourier map
*R*[*F*^2^ > 2σ(*F*^2^)] = 0.032	Hydrogen site location: inferred from neighbouring sites
*wR*(*F*^2^) = 0.074	H-atom parameters constrained
*S* = 1.11	*w* = 1/[σ^2^(*F*_o_^2^) + (0.028*P*)^2^ + 4.815*P*] where *P* = (*F*_o_^2^ + 2*F*_c_^2^)/3
1855 reflections	(Δ/σ)_max_ < 0.001
133 parameters	Δρ_max_ = 1.23 e Å^−^^3^
0 restraints	Δρ_min_ = −1.08 e Å^−^^3^

### (II) catena-Poly[[dichloridomercury(II)]-µ-5,6-bis(pyridin-2-yl)pyrazine-2,3-dicarboxylato-κ2N5:N6] Special details

**Table:**

Geometry. All esds (except the esd in the dihedral angle between two l.s. planes) are estimated using the full covariance matrix. The cell esds are taken into account individually in the estimation of esds in distances, angles and torsion angles; correlations between esds in cell parameters are only used when they are defined by crystal symmetry. An approximate (isotropic) treatment of cell esds is used for estimating esds involving l.s. planes.

### (II) catena-Poly[[dichloridomercury(II)]-µ-5,6-bis(pyridin-2-yl)pyrazine-2,3-dicarboxylato-κ2N5:N6] Fractional atomic coordinates and isotropic or equivalent isotropic displacement parameters (Å^2^)

**Table:**

	*x*	*y*	*z*	*U*_iso_*/*U*_eq_	
Hg1	0.7500	0.64352 (4)	0.7500	0.03521 (13)	
Cl1	0.9248 (2)	0.6841 (2)	0.92787 (14)	0.0574 (5)	
O1	0.5742 (7)	0.9336 (6)	0.8357 (5)	0.0780 (19)	
O2	0.3825 (7)	0.9822 (5)	0.6779 (4)	0.0557 (13)	
N1	0.4215 (6)	0.6897 (5)	0.7942 (4)	0.0319 (11)	
N2	0.5940 (6)	0.4607 (5)	0.8247 (4)	0.0341 (12)	
C1	0.3392 (7)	0.7964 (6)	0.7663 (5)	0.0315 (13)	
C2	0.3366 (7)	0.5809 (6)	0.7789 (4)	0.0279 (12)	
C3	0.4304 (7)	0.4674 (6)	0.8294 (4)	0.0289 (12)	
C4	0.3561 (7)	0.3796 (6)	0.8876 (5)	0.0365 (15)	
H4	0.2428	0.3874	0.8903	0.044*	
C5	0.4514 (9)	0.2810 (7)	0.9412 (5)	0.0467 (17)	
H5	0.4030	0.2207	0.9795	0.056*	
C6	0.6207 (9)	0.2725 (7)	0.9373 (6)	0.0463 (17)	
H6	0.6886	0.2070	0.9729	0.056*	
C7	0.6856 (8)	0.3655 (7)	0.8783 (5)	0.0419 (16)	
H7	0.7995	0.3609	0.8761	0.050*	
C8	0.4467 (9)	0.9113 (7)	0.7670 (6)	0.0432 (16)	
C9	0.4826 (13)	1.0900 (9)	0.6588 (9)	0.084 (3)	
H9A	0.4477	1.1161	0.5817	0.126*	
H9B	0.6003	1.0671	0.6752	0.126*	
H9C	0.4661	1.1581	0.7070	0.126*	

### (II) catena-Poly[[dichloridomercury(II)]-µ-5,6-bis(pyridin-2-yl)pyrazine-2,3-dicarboxylato-κ2N5:N6] Atomic displacement parameters (Å^2^)

**Table:**

	*U*^11^	*U*^22^	*U*^33^	*U*^12^	*U*^13^	*U*^23^
Hg1	0.02386 (18)	0.0439 (2)	0.03582 (19)	0.000	0.00254 (12)	0.000
Cl1	0.0379 (9)	0.0962 (16)	0.0361 (8)	−0.0145 (10)	0.0041 (7)	−0.0109 (9)
O1	0.061 (4)	0.065 (4)	0.090 (4)	−0.029 (3)	−0.022 (3)	0.019 (3)
O2	0.053 (3)	0.045 (3)	0.067 (3)	−0.007 (3)	0.010 (3)	0.018 (3)
N1	0.023 (2)	0.036 (3)	0.037 (3)	−0.003 (2)	0.008 (2)	0.004 (2)
N2	0.025 (2)	0.040 (3)	0.036 (3)	0.000 (2)	0.006 (2)	0.003 (2)
C1	0.031 (3)	0.028 (3)	0.036 (3)	−0.001 (3)	0.008 (2)	0.003 (3)
C2	0.022 (3)	0.037 (3)	0.028 (3)	0.003 (3)	0.011 (2)	0.001 (3)
C3	0.027 (3)	0.031 (3)	0.027 (3)	−0.001 (2)	0.004 (2)	0.000 (2)
C4	0.024 (3)	0.046 (4)	0.039 (3)	−0.002 (3)	0.007 (2)	0.003 (3)
C5	0.048 (4)	0.053 (5)	0.036 (3)	−0.005 (4)	0.002 (3)	0.008 (3)
C6	0.044 (4)	0.044 (4)	0.045 (4)	0.011 (3)	−0.001 (3)	0.014 (3)
C7	0.028 (3)	0.054 (4)	0.042 (3)	0.009 (3)	0.005 (3)	0.010 (3)
C8	0.041 (4)	0.038 (4)	0.051 (4)	0.003 (3)	0.009 (3)	0.003 (3)
C9	0.092 (7)	0.055 (6)	0.105 (8)	−0.020 (5)	0.022 (6)	0.031 (5)

### (II) catena-Poly[[dichloridomercury(II)]-µ-5,6-bis(pyridin-2-yl)pyrazine-2,3-dicarboxylato-κ2N5:N6] Geometric parameters (Å, º)

**Table:**

Hg1—Cl1^i^	2.3464 (16)	C2—C2^ii^	1.420 (11)
Hg1—Cl1	2.3464 (16)	C2—C3	1.480 (8)
Hg1—N2	2.590 (5)	C3—C4	1.389 (8)
Hg1—N2^i^	2.590 (5)	C4—C5	1.373 (9)
Hg1—N1	2.876 (5)	C4—H4	0.9300
O1—C8	1.197 (8)	C5—C6	1.386 (10)
O2—C8	1.327 (8)	C5—H5	0.9300
O2—C9	1.450 (9)	C6—C7	1.393 (10)
N1—C1	1.317 (8)	C6—H6	0.9300
N1—C2	1.334 (8)	C7—H7	0.9300
N2—C7	1.333 (8)	C9—H9A	0.9600
N2—C3	1.342 (7)	C9—H9B	0.9600
C1—C1^ii^	1.409 (12)	C9—H9C	0.9600
C1—C8	1.497 (9)		
			
Cl1^i^—Hg1—Cl1	158.87 (12)	C5—C4—H4	120.3
N2—Hg1—N2^i^	83.1 (2)	C3—C4—H4	120.3
Cl1^i^—Hg1—N2	102.86 (12)	C4—C5—C6	119.1 (7)
Cl1—Hg1—N2	92.97 (12)	C4—C5—H5	120.4
Cl1^i^—Hg1—N2^i^	92.97 (12)	C6—C5—H5	120.4
Cl1—Hg1—N2^i^	102.86 (12)	C5—C6—C7	117.7 (6)
C8—O2—C9	116.6 (6)	C5—C6—H6	121.1
C1—N1—C2	119.4 (5)	C7—C6—H6	121.1
C7—N2—C3	117.7 (5)	N2—C7—C6	123.8 (6)
C7—N2—Hg1	118.5 (4)	N2—C7—H7	118.1
C3—N2—Hg1	122.8 (4)	C6—C7—H7	118.1
N1—C1—C1^ii^	120.0 (3)	O1—C8—O2	125.3 (7)
N1—C1—C8	115.9 (5)	O1—C8—C1	125.0 (7)
C1^ii^—C1—C8	124.0 (4)	O2—C8—C1	109.7 (6)
N1—C2—C2^ii^	119.3 (3)	O2—C9—H9A	109.5
N1—C2—C3	116.4 (5)	O2—C9—H9B	109.5
C2^ii^—C2—C3	124.2 (3)	H9A—C9—H9B	109.5
N2—C3—C4	122.3 (5)	O2—C9—H9C	109.5
N2—C3—C2	116.4 (5)	H9A—C9—H9C	109.5
C4—C3—C2	121.1 (5)	H9B—C9—H9C	109.5
C5—C4—C3	119.4 (6)		
			
C2—N1—C1—C1^ii^	7.6 (10)	C2—C3—C4—C5	−174.7 (6)
C2—N1—C1—C8	−169.7 (5)	C3—C4—C5—C6	1.0 (10)
C1—N1—C2—C2^ii^	7.3 (9)	C4—C5—C6—C7	−0.2 (11)
C1—N1—C2—C3	−170.0 (5)	C3—N2—C7—C6	0.8 (10)
C7—N2—C3—C4	0.1 (9)	Hg1—N2—C7—C6	169.5 (6)
Hg1—N2—C3—C4	−168.1 (4)	C5—C6—C7—N2	−0.7 (11)
C7—N2—C3—C2	174.1 (5)	C9—O2—C8—O1	4.7 (12)
Hg1—N2—C3—C2	5.9 (7)	C9—O2—C8—C1	−173.0 (7)
N1—C2—C3—N2	−38.4 (7)	N1—C1—C8—O1	−39.6 (10)
C2^ii^—C2—C3—N2	144.5 (7)	C1^ii^—C1—C8—O1	143.2 (9)
N1—C2—C3—C4	135.7 (6)	N1—C1—C8—O2	138.2 (6)
C2^ii^—C2—C3—C4	−41.5 (9)	C1^ii^—C1—C8—O2	−39.0 (10)
N2—C3—C4—C5	−1.0 (9)		

Symmetry codes: (i) −*x*+3/2, *y*, −*z*+3/2; (ii) −*x*+1/2, *y*, −*z*+3/2.

### (II) catena-Poly[[dichloridomercury(II)]-µ-5,6-bis(pyridin-2-yl)pyrazine-2,3-dicarboxylato-κ2N5:N6] Hydrogen-bond geometry (Å, º)

**Table:**

*D*—H···*A*	*D*—H	H···*A*	*D*···*A*	*D*—H···*A*
C9—H9*A*···Cl1^iii^	0.96	2.81	3.647 (9)	146

Symmetry code: (iii) *x*−1/2, −*y*+2, *z*−1/2.

## References

[bb1] Alfonso, M. (1999). PhD thesis, University of Neuchâtel, Switzerland.

[bb2] Alfonso, M. & Stoeckli-Evans, H. (2016). *Acta Cryst.* E**72**, 233–237. 10.1107/S2056989016001080 PMC4770964 26958396

[bb3] Alfonso, M., Wang, Y. & Stoeckli-Evans, H. (2001). *Acta Cryst.* C**57**, 1184–1188. 10.1107/s0108270101010757 11600779

[bb4] Groom, C. R., Bruno, I. J., Lightfoot, M. P. & Ward, S. C. (2016). *Acta Cryst.* B**72**, 171–179. 10.1107/S2052520616003954 PMC4822653 27048719

[bb5] Lin, M.-J., Jouaiti, A., Kyritsakas, N. & Hosseini, M. W. (2010). *Chem. Commun.* **46**, 115–117. 10.1039/b916249f 20024311

[bb6] Macrae, C. F., Bruno, I. J., Chisholm, J. A., Edgington, P. R., McCabe, P., Pidcock, E., Rodriguez-Monge, L., Taylor, R., van de Streek, J. & Wood, P. A. (2008). *J. Appl. Cryst.* **41**, 466–470.

[bb7] Sheldrick, G. M. (2008). *Acta Cryst.* A**64**, 112–122. 10.1107/S0108767307043930 18156677

[bb8] Sheldrick, G. M. (2015). *Acta Cryst.* C**71**, 3–8.

[bb9] Spek, A. L. (2009). *Acta Cryst.* D**65**, 148–155. 10.1107/S090744490804362X PMC2631630 19171970

[bb10] Stoe & Cie (1997). *STADI4* and *X-RED*. Stoe & Cie GmbH, Damstadt, Germany.

[bb11] Stoe & Cie (2004). *IPDS-I Bedienungshandbuch*. Stoe & Cie GmbH, Darmstadt, Germany.

[bb12] Tadjarodi, A., Bijanzad, K. & Notash, B. (2012). *Acta Cryst.* E**68**, m1099. 10.1107/S1600536812032126 PMC3414149 22904756

[bb13] Westrip, S. P. (2010). *J. Appl. Cryst.* **43**, 920–925.

[bb14] Yang, L., Powell, D. R. & Houser, R. P. (2007). *Dalton Trans.* pp. 955–964. 10.1039/b617136b 17308676

[bb15] Zhang, G.-N. (2008). *Acta Cryst.* E**64**, m357. 10.1107/S1600536808000986 PMC2960254 21201316

